# Spatial and seasonal group size variation of wild mammalian herbivores in multiple use landscapes of the Ngorongoro Conservation Area, Tanzania

**DOI:** 10.1371/journal.pone.0267082

**Published:** 2022-04-19

**Authors:** Cecilia M. Leweri, Gundula S. Bartzke, Maurus J. Msuha, Anna C. Treydte

**Affiliations:** 1 Department of Sustainable Agriculture, Biodiversity and Ecosystem Management, School of Life Sciences and Bio-Engineering, The Nelson Mandela African Institution of Science and Technology, Arusha, Tanzania; 2 Wildlife Information and Education Unit, Tanzania Wildlife Research Institute (TAWIRI), Arusha, Tanzania; 3 Institute of Crop Science, Biostatistics, University of Hohenheim, Stuttgart, Germany; 4 Wildlife Division, Ministry of Natural Resources and Tourism, Dodoma, Tanzania; 5 Hans Ruthenberg Institute, Ecology of Tropical Agricultural Systems, University of Hohenheim, Stuttgart, Germany; 6 Department of Physical Geography, Stockholm University, Stockholm, Sweden; Sichuan University, CHINA

## Abstract

Group sizes of wild herbivores can be indicators of ecosystem health and proxies for individual and population fitness, particularly in areas where human activities have become increasingly common. We recorded 176 single- and multi-species groups of wild herbivores in human-dominated landscapes of the Ngorongoro Conservation Area (NCA) during dry and wet seasons. We analyzed how wild herbivore group sizes were affected by: (1) season, (2) distance to fully protected area (NCA crater) and to streams, (3) distance to human settlements, and (4) numbers of livestock. Group sizes were generally larger during the wet season than during the dry season and varied seasonally with distance to NCA crater, streams, and human settlements. During the wet season, larger groups were observed further away from the NCA crater whereas the opposite pattern was apparent during the dry season. Average wild herbivore group sizes increased by about three-fold with increasing distance from the streams during the dry season but were invariant to streams during the wet season. Furthermore, during the dry season, group sizes were larger close to settlements but varied little with distance to settlements during the wet season. While livestock presence did not directly affect wild herbivore group size, distance to settlements, streams and distance to the Ngorongoro crater in interaction with rainfall seasonality did. We conclude that the NCA crater functions as a key resource area for wild herbivores such as wildebeest (*Connochaetes taurinus*) and zebra (*Equus quagga burchelli*) during the dry season, highlighting the need for its full protection status in this Man and Biosphere reserve.

## Introduction

Group size and composition are the most basic elements of social organization for ungulates living in herds as they influence both foraging, migration, and other daily activities [1]. Theoretical frameworks explaining variation in group size assume that there is a trade-off between fitness relevant costs and benefits, and that individuals maintain membership in groups of optimal sizes to maximize fitness [2, 3]. In some African ungulates, for example, group sizes and their spatial distributions vary temporarily with season as rainfall governs the quantity and quality of vegetation [4, 5]. Despite the significance of long-term population monitoring and studies on population dynamics and movements [6–8], the latter have not yet addressed the effects of human presence and livestock grazing on herbivore group sizes in pastoral and protected areas for conservation planning [9].

Humans and livestock increase pressure on rangelands, and add to the complexity of their management, especially in areas where wild herbivores strongly interact with livestock on a daily basis [10, 11]. Interactions between livestock and wildlife can be either competitive or facilitative, depending on the species involved, and on the seasonal availability of resources [12, 13]. For example, wild herbivores have coexisted with domestic herbivores in few subsistence pastoral systems with abundant water points [13, 14]. However, high livestock densities can also outcompete wild herbivores [15] and reduce wild herbivore group sizes [9, 16] or lead to long-term declines in the abundance and diversity of native wildlife [17, 18]. Competition often occurs during the dry season, when grazing ranges are constricted near available water resources and when overall fodder quality is lower than during the wet season [16]. A consecutive group size reduction may impact reproductive fitness of wild herbivores and, hence, their population dynamics [2, 19]. With an increasing growth of human and livestock population, there is a pressing need for research concerning the ecology and management of wild herbivores, particularly their group sizes and behavior in response to human presence and to changing environmental conditions.

Landscape features such as elevation, seasonal streams and natural vegetation could also alter wild herbivores group sizes [20–22] since they determine the quality and structure of habitats and likely the distribution of food patches [19, 23]. Surface water for example is well documented to attract wild animals [15, 24]. Elevation is another variable that can alter herbivore group sizes as plant biomass often decreases with elevation [25, 26]. Understanding how different landscape features influence group sizes can therefore inform decisions in species conservation and management, such as identifying conservation areas [27–29].

Various studies on herbivores analyzed the long-term population trends and assessed the seasonal stability of wild herbivore communities in the Ngorongoro Conservation Area (NCA) [30]. In addition, some studies found that the exclusion of resident pastoralists and their livestock from the Ngorongoro crater resulted into both increasing and decreasing trends of some ungulate species whereas overall, wildlife biomass remained unchanged [31, 32]. However, as humans continue to impact natural habitats and livestock populations still grow within NCA, there is an increasing need to understand how this affects distributions and group sizes of wild herbivores in this Man and Biosphere reserve [33, 34]. We, therefore, assessed how wild herbivore group sizes respond to environmental and anthropogenic variables such as livestock keeping, and human settlements in the NCA, and whether this response varies between season.

We hypothesized that larger wildlife groups will be formed during the wet season than during the dry season, i.e., when forage is abundant. We also expected that larger groups will be formed closer to the streams due to the higher availability of water and food compared with areas further away from streams [35]. Furthermore, we expected that groups will be larger in areas of low competition with livestock herds. In addition, we hypothesized that wildlife groups will be larger further away from settlements (houses and livestock enclosure or temporary corrals, in which livestock are herded at night to protect them against predators).

## Material and methods

### Study area

**Fig 1:**
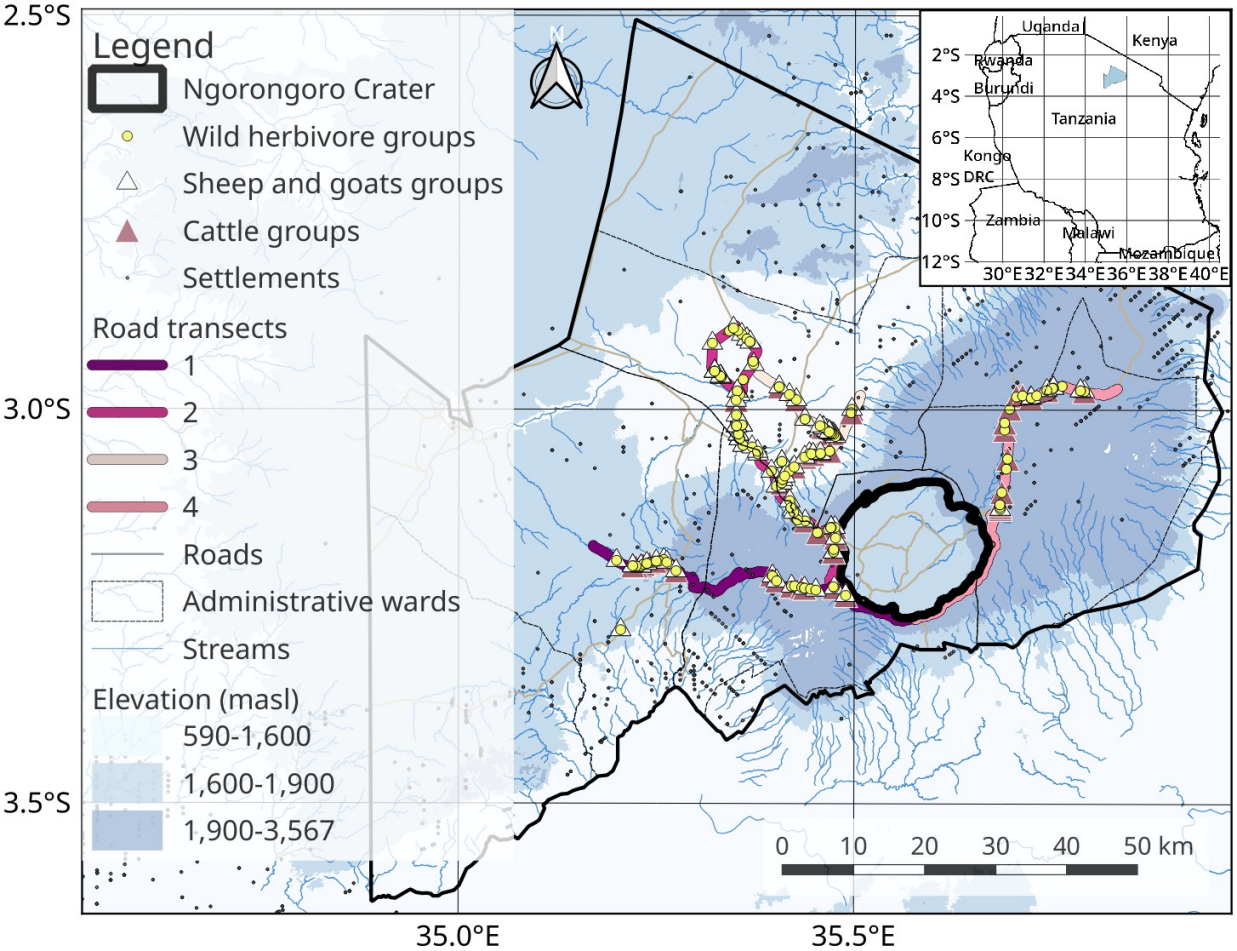
Map of Ngorongoro Conservation Area (NCA) in northern Tanzania showing locations of wild and domestic (cattle, sheep and goats) herbivore groups. In addition, we show the location of settlements that had been observed along road transects in the four wards Nainokanoka, Ngorongoro, Endulen and Olbalbal, from May 2018 to February 2019. (Source of dataset is provided in Table 1).

We conducted our study in four wards of the Ngorongoro Conservation Area (NCA), a UNESCO World Heritage Site in Northern Tanzania (3°14’29.56"S and 35°29’16"E; Fig 1) with a total size of 8,256 km^2^ [36]. This area extends over part of the Great Rift Valley of East Africa and contains grassland plains, savanna woodlands, forests, mountains, volcanic craters, lakes, rivers, and swampland. Ecologically, NCA is categorized into three zones; lowlands, midlands, and highlands [37], and its climatic zones span from semi-arid to montane forest climate, with average annual precipitation ranging from less than 500 mm up to 1,700 mm [38]. A description of the sources of the dataset used to produce a map in Fig 1 is provided in Table 1.

**Table 1:** Source of the datasets used to produce the map of the Ngorongoro Conservation Area, northern Tanzania.

Data sets	Institution	Citation and Website
Protected Areas boundaries	Serengeti GIS and Data Center	[41] https://serengetidata.weebly.com/boundaries.html Accessed 2021-10-17
Administrative wards	Tanzania National Bureau of Statistics	https://www.nbs.go.tz/index.php/en/census-surveys/population-and-housing-census/173-2012-phc-shapefiles-level-three Accessed 2022-03-17
Streams	Serengeti GIS and Data Center	[42] https://serengetidata.weebly.com/rivers-and-lakes.html Accessed 2021-10-18
Road	Socioeconomic Data and Applications Center (SEDAC)	[43] https://sedac.ciesin.columbia.edu/data/set/groads-global-roads-open-access-v1 Accessed 2021-10-18
Elevation raster file	USGS	[44] https://doi.org/10.5067/MEaSUREs/SRTM/SRTMGL1.003 Accessed 2022-03-18
Settlements	Tanzania Wildlife Research Institute (TAWIRI)	https://tawiri.or.tz/ Accessed 2021-10-18

Rainfall in NCA is highly seasonal and spatially variable. The eastern slopes of the crater highlands receive average annual rainfall of about 1,200 mm/year, whereas the midlands receive about 800 mm/year and the lowlands receive only 400 mm/year [39]. Average annual temperatures lie between 2°C and 35°C [38]. We selected four wards: Nainokanoka (average elevation is 2,440 masl), Ngorongoro (1,996 masl), Endulen (1,637 masl) and Olbalbal (1,548 masl) for our field study to cover a large elevational gradient and varying distances to the fully protected area, i.e., the NCA crater (Fig 1). The area covered by each elevation belt was 1,567 km^2^ for lowlands (less than 1,600 masl): 1,819 km^2^ for midlands (1,600–1,900 masl): and 1,789 km^2^ for highlands (above 1,900 masl). The NCA crater fully excludes pastoralists and their livestock herds, whereas other parts of the NCA are shared by both pastoralists and wildlife. The Maasai are the dominant pastoralists in NCA, they live in settlements called boma or a homestead, which is a grouping of houses of multiple families enclosed by thorny *Acacia* branches to deter predators from entry [40].

The main economic activities in the NCA are livestock keeping and tourism [45]. The livestock species are cattle (*Bos taurus*), goats (*Capra aegagrus hircus*), sheep (*Ovis aries*) and donkeys (*Equus asinus*). The dominant wild mammalian herbivore species include plains zebra (*Equus quagga burchelli*), common eland (*Tragelaphus oryx*), blue wildebeest (*Connochaetes taurinus*), African buffalo (*Syncerus caffer*), Grant’s gazelle (*Nanger* g*ranti*), and Thomson’s gazelle (*Eudorcas thomsonii*). Less common species in the NCA are the giraffe (*Giraffa camelopardalis*), black rhinoceros (*Diceros bicornis*) and African elephant (*Loxodonta africana*) [46].

### Data collection

Group sizes of wild herbivores were recorded during four sampling periods, two in each season (wet season: November–May, dry season: June–October) between May 2018 and February 2019. Four roads were chosen as transects. Each of those transects was visited four times during the entire study period. Transect 1 covered 55.8 km length and was distributed across an average (± SD) elevation of 2,097 ± 288 masl; transect 2 covered 68.1 km and 1,659 ± 281 masl; transect 3 covered 35.9 km, and 1,337 ± 58 masl; and transect 4 covered 56.5 km and 2,448 ± 90 masl. Roads were repetitively sampled using the road strip census method, in which animals were counted from the car within a certain strip width [47]. We drove the car at a constant speed of 25 km/h for 6 h each day, 3 h in the morning (07:00–10:00 h) and 3 h in the evening (15:30–18:30 h).

Observations of wild mammalian herbivore groups were restricted to distances within 250 m from the road to ensure visibility. For each sighting, we recorded the GPS coordinates and counted the number of individuals in the group of wild herbivores and livestock (defined as individuals within 50 m of each other). We used a rangefinder (Bushnell Elite 1500) to measure the perpendicular distance between the centre of each animal group and the observer [48]. The distances of all observed wild mammalian herbivore groups to the NCA crater rim (i.e., fully protected area), to the nearest settlement (i.e., human influence) and to the nearest stream (environmental factor) were obtained using QGIS version 3.6.

As the sample sizes for group observations of some species were rather low (S1 Fig) we categorized the wild herbivores into browsers (giraffe), grazers (zebra, wildebeest, and buffalo), or mixed feeders (Grant’s gazelle, Thompson’s gazelle and eland) [49]. We further omitted two outlying observations of an unexpectedly large Thompson’s gazelle group and one elephant group. Livestock groups were categorized as either cattle or the combination of sheep and goats, due to the difficulty in distinguishing between sheep and goats in large mixed herds.

### Data processing

We recorded wild herbivore group sizes across various village areas of the Ngorongoro Conservation Area (NCA) during the wet and dry season. We combined field-based techniques documenting locations and group sizes of wild herbivores and livestock with settlements locations collected by the Tanzania Wildlife Research Institute (TAWIRI) during an aerial census in the year 2016 [50]. Furthermore, we analyzed if wild herbivore group sizes were affected by (1) season, given the local climatic projections of greater rainfall variability, both within and between seasons [51, 52]; (2) landscape features such as distance to the fully protected area, i.e., the NCA crater, and distance to streams; (3) distance to human settlements; and (4) the number of livestock individuals present in proximity to the wildlife groups. A description of the range of variables used to model the group sizes of wild herbivores are listed in Table 2.

**Table 2:** Range of variables used to model the group sizes of wild herbivores in response to environmental variables, human settlements and livestock in the Ngorongoro Conservation Area, northern Tanzania, from May 2018 to February 2019.

Variable Name	Category	Data range (min–max)
Distance to streams (km)	environment	0.0–7.5
Distance to the NCA crater (km)	environment	0.3–31.7
Elevation (masl)	environment	1,288–2,654
Dry season versus wet season	environment	
Distance to the nearest settlement (km)	human	0.1–5.6
Number of cattle in proximity to wild herbivores	livestock	1–250
Number of sheep and goats in proximity to wild herbivores	livestock	1–842

### Data analyses

A generalized linear mixed model (GLMM) was applied to analyse the potential effects of season (wet vs dry), distance to the NCA crater, distance to streams (seasonal rivers in the ecosystem), elevation, livestock herds and distance to settlements on group sizes of wild herbivores. To account for repeated samples from the same transects, we nested transects in seasons and included them as a random factor. We further included the sampling date as a random factor [53]. All pair-wise correlation coefficients metric ranged from -0.4 to 0.4, indicating low levels of co-linearity [54]. We applied a zero-truncated negative binomial regression model because the observed group sizes were always larger than zero and the empirical histogram indicated that the data was strongly over-dispersed [55]. The positive negative binomial distribution was given by

$$f\left(y_{i};k,\mu_{i}|y_{i}>0\right)=\frac{\frac{ᴦ(y_{i}+k)}{ᴦ(k)\times ᴦ(y_{i}+1)}\times ({\frac{k}{\mu_{i}+k})}^{k}\times {\left(1-\frac{k}{\mu_{i}+k}\right)}^{y_{i}}}{\left(1-({\frac{k}{\mu_{i}+k})}^{k}\right)},$$

where *y*_*i*_ are the *i = 1*, *2*,*…*, *n*_*i*_ observed wild herbivore group sizes, *Γ* is the gamma function, *μ*_*i*_ is the mean of the ordinary binomial distribution and *k* is the dispersion parameter [55, 56].

Our initial model (Eq. 1 in S2 Text) was based on the theory that variation in the environment, human activities, and competition with livestock affect the availability of resources that enable wild herbivores to form groups [57]. We incorporated interactions between season and human, environmental and livestock variables in the initial model because we expected that these covariate effects may vary seasonally and accounted for group size differences between feeding guilds (S1 Table). The perpendicular distances of animal groups to the observer were also included as an explanatory variable because herbivore group sizes could have been affected by the presence of a vehicle and closeness to roads (Eq. 1 in S2 Text).

Backwards selection of variables [58], using the drop1 function in R version 3.6.1 [59], as further used to select the most influential variables using Likelihood Ratio (LR) tests [60]. Variables were deleted from the full model starting with interaction and main effects of the variables with the highest *P*-values until all remaining variables had *P*-values of 0.056 or below. Although “statistical significance” is often interpreted as *P* < 0.05, we kept seasonal interaction terms with stream distances (*P* = 0.056) in the final model as water availability is an important variable in conservation planning [61]. Throughout the process, we kept distance to the observer as a confounding variable in the model.

During the selection procedure, seasonal interaction effects for sheep and goats were eliminated followed by the main effect of sheep and goats, seasonal interaction effects for cattle and the main effect of cattle (Eqs. 2–5 in S1 Text and S2–S5 Tables). The last variables to be eliminated were seasonal elevation effects and the main effect of elevation (Eqs. 6 and 7 in S2 Text and S6 and S7 Tables).

We predicted wild herbivore group sizes based on the reduced model (Eq. 7 in S2 Text) in relation to the environmental, human and livestock variables for each feeding guild and season. Post hoc Tukey HSD Pairwise comparisons were applied for group size differences between feeding guilds. The zero-truncated negative binomial regression models were implemented via the glmmTMB R-package.

## Results

We observed 176 groups of wild mammalian herbivores, with more observations (98; 56%) during the wet season than during the dry season (78; 44%). Wild herbivore group sizes were often larger in the wet season than in the dry season. Of all observed groups, 74% were formed by grazers, 18% by mixed feeders, and 8% by browsers. Grazers had similar group sizes as mixed feeders (t = -0.02, df = 161, *P* = 0.999) whereas browsers had group sizes that were by about 1/3 smaller than both grazers (t = -4.02, df = 161, *P* < 0.001) and mixed feeders (t = -3.42, df = 161, *P* = 0.002) (S2 Fig).

### Larger groups closer to NCA crater in the dry season

**Fig 2:**
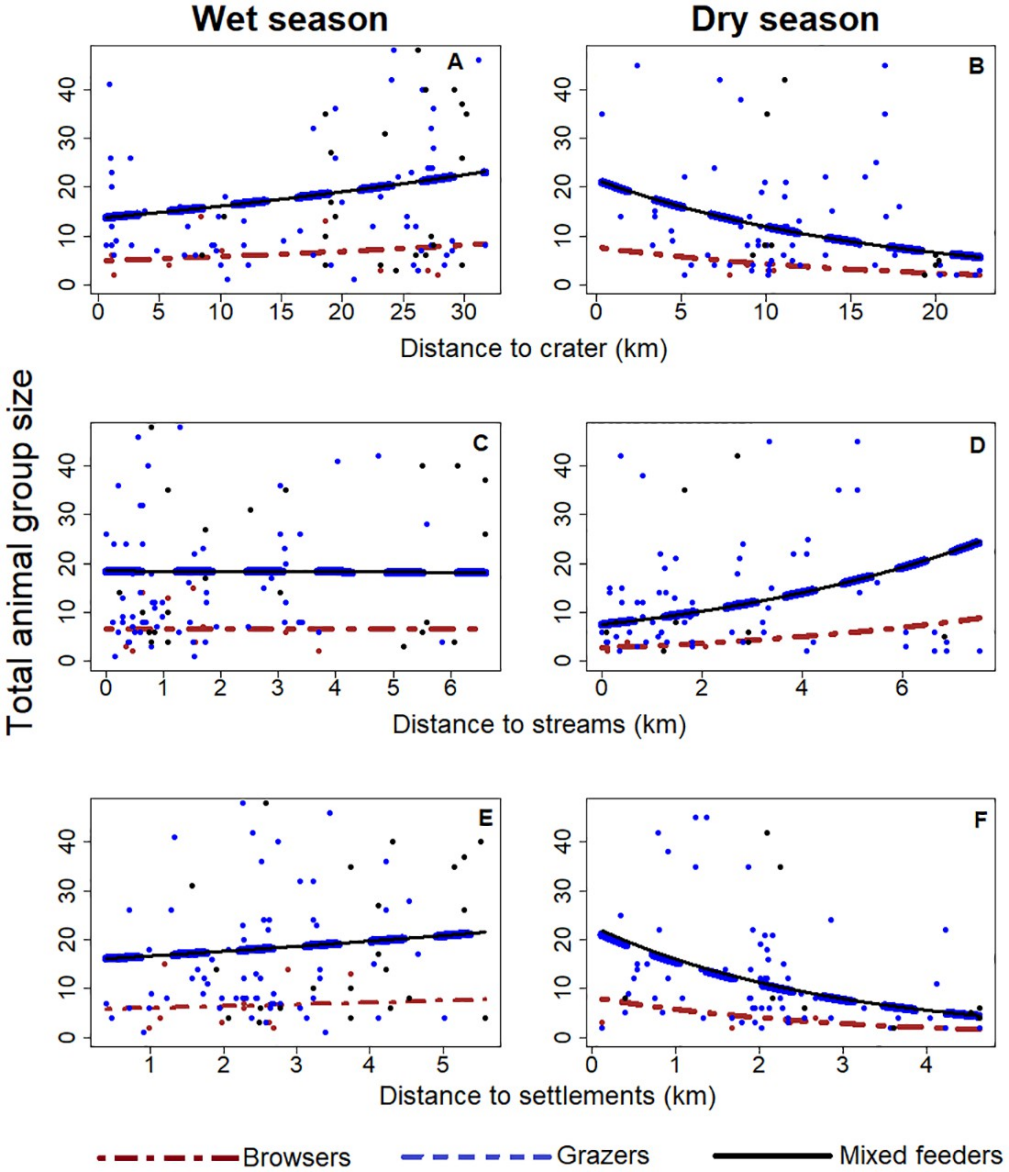
Trends in wild herbivore group sizes for browsers (giraffe), grazers (zebra, wildebeest, and buffalo) and mixed feeders (Grant’s gazelle, Thompson’s gazelle and eland) in relation to the fully protected area of the Ngorongoro crater (A, B), distance from streams (C, D), and settlements (E, F) during the wet season (left panels) and dry season (right panels) in the Ngorongoro Conservation Area, northern Tanzania, from May 2018 to February 2019.

Wild mammalian herbivore group sizes varied seasonally with distance away from the NCA crater (LR-test_1, 2_ = 10.5, *P* = 0.001). During the wet season, group sizes doubled from about 4 browsers at the NCA crater rim to 8 browsers at a distance of 32 km away from the crater (Fig 2A). Similarly, an average group size of 14 grazers and mixed feeders at the crater rim doubled to a group size of 23 grazers and mixed feeders at 32 km away from the crater rim (Fig 2A). In contrast, during the dry season, the estimated group sizes decreased about three-fold with increasing distance away from the NCA crater, i.e., from 8 browsers, 21 grazers and mixed feeders at the crater rim to 2 browsers, 6 grazers and 6 mixed feeders at 23 km distance away from the NCA crater (Fig 2B).

### Larger groups further away from streams in the dry season

Wild herbivore group sizes varied across season with distance to streams (LR-test _1, 2_ = 3.7, *P* = 0.056). Most groups (67%) were observed at less than 2 km distance to streams, but group sizes did not change in relation to distance from the streams during the wet season, generally occurring from 0 to 8 km distance away from streams (Fig 2C). During the dry season, more than half of all herbivore groups (56%) were observed at less than 2 km distance to streams. But estimated group sizes increased by about three-fold with increasing distance from the streams, i.e., from 2 browsers, 7 grazers and 7 mixed feeders next to streams to 8 browsers, 24 grazers and 25 mixed feeders at 8 km away from streams (Fig 2D).

### Larger groups close settlements

Wild herbivore groups responded differently to the presence of settlements in each season (LR-test _1, 2_ = 8.5, *P* = 0.004). During the wet season, wild herbivore group sizes were slightly higher closer to settlements than further, i.e., about 6 km, away (Fig 2E). However, during the dry season, group sizes decreased from 8 to 1 browser, 21 to 4 grazers and 22 to 6 mixed feeders with increasing distance away up to about 5 km away from settlements (Fig 2F).

## Discussion

Our results on fluctuating group sizes of large wild herbivores in the Ngorongoro Conservation Area indicate that pastoralist activities and environmental variables affect the social organization of ungulates [10, 62]. These activities imply that animals have to spend more time being vigilant and have less time available for feeding [63–65]. Higher vigilance has potential consequences for reproductive fitness [66, 67], will reduce species abundance and alter community structure [68]. The tendency of increased wild herbivore group sizes we observed during the wet season agrees with results from another study [69, 70] that attributed the group size change to increased food availability.

During the wet season, wild herbivore group sizes increased further away from the NCA crater, possibly because herbivores disperse into short grass plains maintained by livestock grazing [40]. There is potential benefit from facilitation by livestock for wild herbivores, i.e., providing short‐grass patches of high forage quality for growth and reproduction of herbivore populations [46, 71]. Our findings that wild herbivore group sizes increased closer to the NCA crater during the dry season is likely due to limited food availability further away from the NCA crater, triggered by competition with livestock further away from the fully protected area [72]. The NCA crater rim contains various shrubs and flowering plants [40] that may attract herbivores during the dry season [73, 74]. These spatial changes in group sizes might indicate a significant collective movement pattern towards the NCA crater potentially in search for food resources [75–77].

Moreover, the permanently flowing rivers inside the crater may attract animals in times of low rainfall, and particularly wildebeest, African buffalo, African elephant and zebra [78–81]. However, against our expectations we did not detect a humped-shaped distribution of wild herbivores with distance to water, contrary to other studies [15]. Our observed aggregation of herbivore groups close to streams in the dry season may indicate that herbivores were attracted to water. But increased competition and changes in vegetation resulting from trampling by herbivores and livestock might have influenced the herbivores’ ability to form large groups [82–84].

Several studies suggest that associations between wild and domestic herbivores with similar body size and niche would lead to competitive exclusion of the wild herbivores [85, 86]. Contrary to our expectations, changes in wild herbivore group sizes were better explained by variations in the environmental and human settlements rather than by the presence of livestock. Whenever we saw livestock and wildlife together, there was no direct aggression or replacement by livestock (personal observations). On the other hand, resource use by Maasai cattle in NCA closely resembles that of resident wildlife [12] and diets of cattle and wild herbivores including impala, plain zebra, and wildebeest overlap in East African savannas [87]. However, evidence for competition between livestock and wild herbivores are scarce [9] except for few studies, which observed less abundance of wild herbivores within a radius of 10 km away from human settlements [4].

The increase in wild herbivore group sizes we observed closer to settlements implies that wild herbivores in NCA may have no choice but to aggregate close to settlements during the dry season to use water and food available at these sites. At NCA, as in other rangeland areas of eastern Africa, pastoral movement is largely governed by efforts to find the best pastures, though mostly constrained by the limited distribution of water in the dry season [88–90]. Emerging evidence also suggests a high concentration of wild herbivores in proximity to settlements to avoid predators [15].

## Conclusion

Against our expectations, we found that livestock did not significantly influence wild herbivore group sizes in the multiple use landscape of the Ngorongoro Conservation Area but rather, combined biotic and abiotic factors. Our results reveal how season, distance to protected areas, and distance to streams interactively shape herbivore group sizes in a multiple land use area. However, the observed decline in group sizes further away from the NCA crater during the dry season may suggest that wild herbivores had fewer resources (food and water) available as a result of land use by humans and their livestock. Our study is one of the few in this iconic Man and Biosphere reserve that quantified how human-driven factors might impact seasonal group sizes of wild herbivores. Our work only represents a snapshot over two seasons and we, therefore, recommend long-term monitoring of wild herbivore group sizes. This can provide a valuable indicator of temporal dynamics in wild herbivore group sizes given the ever increasing and poorly managed human and livestock population in the Ngorongoro Conservation Area. These research results can potentially guide alternative approaches to rangeland conservation practices.

## Supporting information

S1 Fig The total number of groups observed per wild and domestic mammalian herbivore species during the wet and during the dry season in the Ngorongoro Conservation Area, northern Tanzania, from May 2018 to February 2019. (TIF)

S2 Fig Mean ± SE of group sizes of wild herbivores browsers (giraffe), grazers (zebra, wildebeest, and buffalo) and mixed feeders (Grant’s gazelle, Thompson’s gazelle and eland) observed along road transects in the Ngorongoro Conservation Area, northern Tanzania, from May 2018 to February 2019. Boxes with the same letters do not differ significantly based on Tukey’s HSD test at *P =* 0.05.
(TIF)

S1 Data Wild herbivore group sizes recorded in Ngorongoro Conservation Area, northern Tanzania, from May 2018 to February 2019. (XLS)

S1 Text R code used to analyse the spatial and temporal distribution of wild mammalian herbivores in Ngorongoro Conservation Area from May 2018 to February 2019. (TXT)

S2 Text Variable selection steps for analyzing wild herbivore group sizes recorded in Ngorongoro Conservation Area, northern Tanzania, from May 2018 to February 2019 in relation to feeding guilds, environmental and human variables, and livestock. (DOCX)

S1 Table Results of Generalized Linear Mixed Model (GLMM) of the initial model (S1 Text, Eq. 1) of wild herbivore group sizes recorded in Ngorongoro Conservation Area, northern Tanzania, from May 2018 to February 2019 in relation to feeding guilds, environmental and human variables, and livestock. (Main effects of season, distances to streams, crater and settlements, sheep and goats, cattle and elevation are not shown).
(XLSX)

S2 Table Results of Generalized Linear Mixed Model (GLMM) after eliminating the seasonal interaction effect for sheep and goats (S1 Text, Eq. 2) of wild herbivore group sizes recorded in Ngorongoro Conservation Area, northern Tanzania, from May 2018 to February 2019 in relation to feeding guilds, environmental and human variables, and livestock. (Main effects of season, distances to streams, crater and settlements, cattle and elevation, are not shown).
(XLSX)

S3 Table Results of Generalized Linear Mixed Model (GLMM) after eliminating the main effect for sheep and goats (S1 Text, Eq. 3) of wild herbivore group sizes recorded in Ngorongoro Conservation Area, northern Tanzania, from May 2018 to February 2019 in relation to feeding guilds, environmental and human variables, and livestock. (Main effects of season, distances to streams, crater and settlements, cattle and elevation are not shown).
(XLSX)

S4 Table Results of Generalized Linear Mixed Model (GLMM) after the eliminating the seasonal interaction effects for cattle (S1 Text, Eq. 4) of wild herbivore group sizes recorded in Ngorongoro Conservation Area, northern Tanzania, from May 2018 to February 2019 in relation to feeding guilds, environmental and human variables, and livestock. (Main effects of season, distances to streams, crater and settlements and elevation are not shown).
(XLSX)

S5 Table Results of Generalized Linear Mixed Model (GLMM) after the eliminating the main effect for cattle (S1 Text, Eq. 5) of wild herbivore group sizes recorded in Ngorongoro Conservation Area, northern Tanzania, from May 2018 to February 2019 in relation to feeding guilds, environmental and human variables. (Main effects of season, distances to streams, crater and settlements and elevation are not shown).
(XLSX)

S6 Table Results of Generalized Linear Mixed Model (GLMM) after the eliminating the seasonal interaction effects for elevation (S1 Text, Eq. 6) of wild herbivore group sizes recorded in Ngorongoro Conservation Area, northern Tanzania, from May 2018 to February 2019 in relation to feeding guilds, environmental and human variables. (Main effects of season, distances to streams, crater and settlements are not shown).
(XLSX)

S7 Table Results of Generalized Linear Mixed Model (GLMM) after the eliminating the main effect for elevation (S1 Text, Eq. 7) of wild herbivore group sizes recorded in Ngorongoro Conservation Area, northern Tanzania, from March 2018 to February 2019 in relation to feeding guilds, environmental and human variables. (Main effects of season, distances to streams, crater, and settlements are not shown).
(XLSX)
