# Supplementary figures and images for: Spatial and seasonal group size variation of wild mammalian herbivores in multiple use landscapes of the Ngorongoro Conservation Area, Tanzania
Source: PLoS One. 2022 Apr 19;17(4):e0267082. doi: 10.1371/journal.pone.0267082 (PMC9017940; doi:10.1371/journal.pone.0267082)

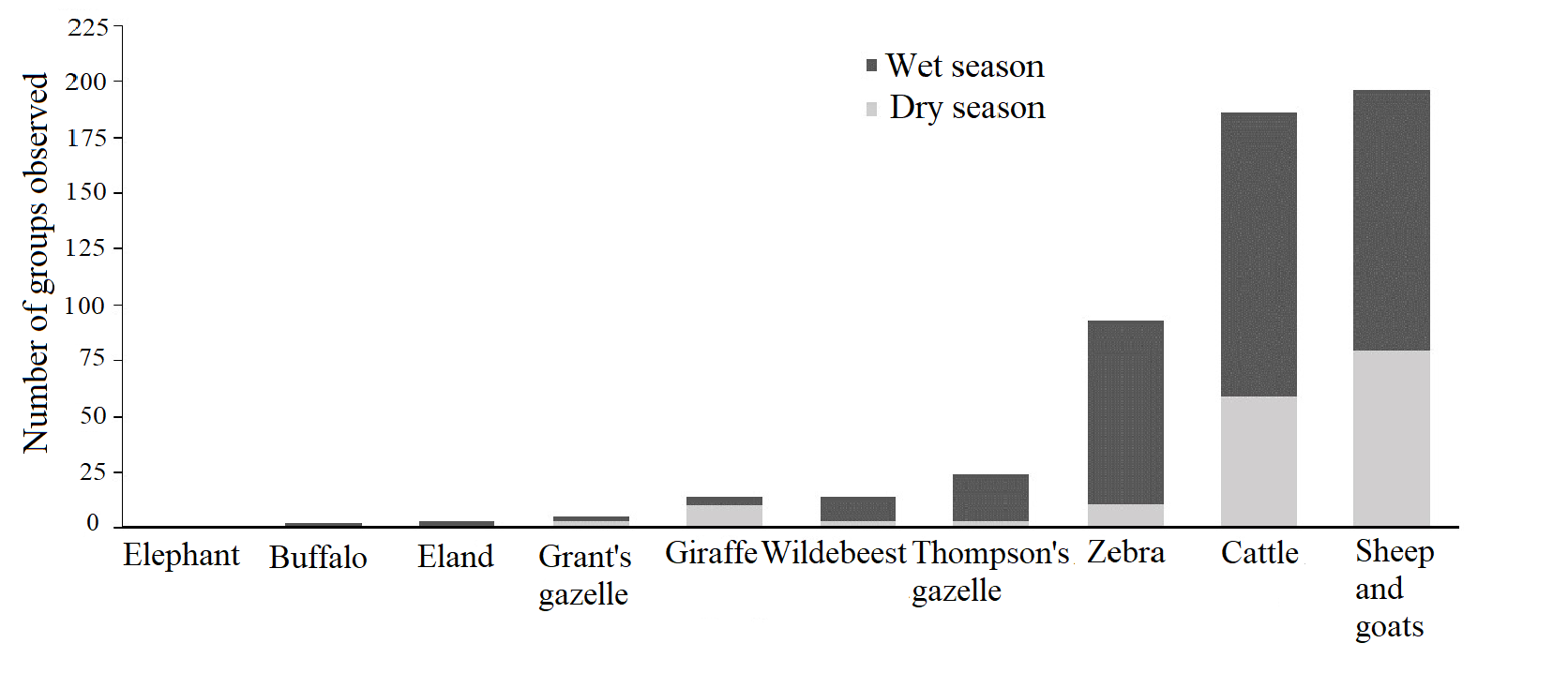

Supplement: S1 Fig — (TIF) [file pone.0267082.s001.tif]

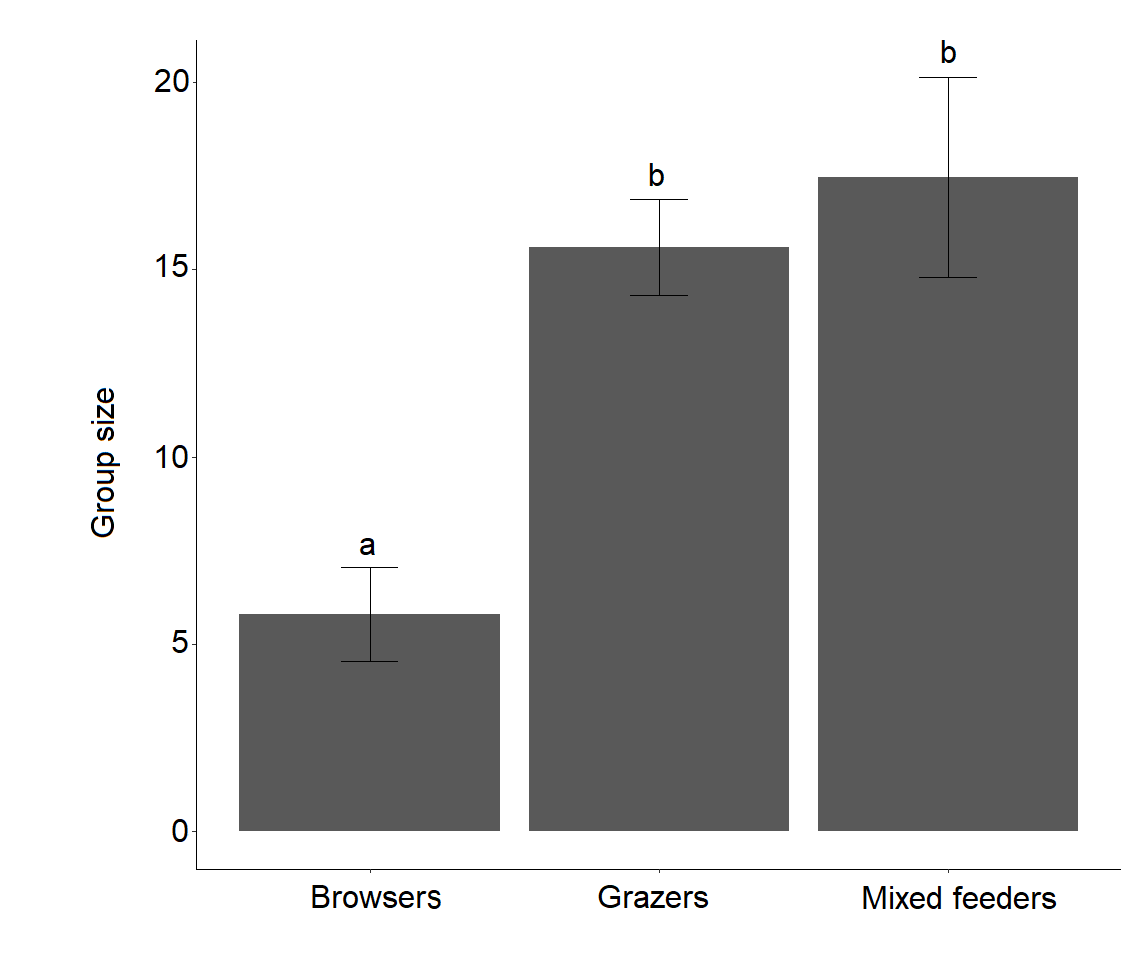

Supplement: S2 Fig — Boxes with the same letters do not differ significantly based on Tukey’s HSD test at P = 0.05. (TIF) [file pone.0267082.s002.tif]
