# Supplementary material for: Spatial and seasonal group size variation of wild mammalian herbivores in multiple use landscapes of the Ngorongoro Conservation Area, Tanzania
Source: PLoS One. 2022 Apr 19;17(4):e0267082. doi: 10.1371/journal.pone.0267082 (PMC9017940; doi:10.1371/journal.pone.0267082)
Supplement: S2 Text — (DOCX) [file pone.0267082.s005.docx]

**Initial model**

The parameter ***μ*** of the zero-truncated negative binomial model (Eq. 1 in main manuscript) was linked to explanatory variables using the logarithmic link function.

${\ln(\mu}_{ijklm})=\eta_{ijklm}=\alpha{+ g}_{j}+s_{k}+\beta_{1}\times x_{i1}{+ \beta_{2}\times x_{i2}+\gamma}_{2k}\times x_{i2}+{\beta_{3}\times x_{i3}+\gamma}_{3k}\times x_{i3}+{\beta_{4}\times x_{i4}+\gamma}_{4k}\times x_{i4}+{\beta_{5}\times x_{i5}+\gamma}_{5k}\times x_{i5}+{\beta_{6}\times x_{i6}+\gamma}_{6k}\times x_{i6}+{\beta_{7}\times x_{i7}+\gamma}_{7k}\times x_{i7}+t_{kl}+d_{m,}$ (1)

where $\eta_{ijklm}$ is the linear predictor for the *i*’th group size of the *j*’th feeding guild in the *k*’th season on the *l*’th transect and *m*’th date. The symbol *α* denotes the general mean, *g_j_* is the effect of the *j*’th feeding guild, *s_k_* the effect of the *k*’th season, *β_1_* is the regression coefficient for distance to observer *x_i1_*, *β_2_* is the regression coefficient for distances to the crater *x_i2,_ β_3_* is the regression coefficient for distances to streams *x_i3_*, *β_4_* is the regression coefficient for distances to settlements *x_i4_*, *β_5_* is the regression coefficient for elevation *x_i5_*, *β_6_* is the regression coefficient for numbers of cattle *x_i6_*, *β_7_* is the regression coefficient for numbers of sheep and goats *x_i7_*, and *γ_1k_, γ_2k,_ ..., γ_7k_* are the corresponding seasonal interaction regression coefficients for the metric variables *x_i1_*, *x_i2_*, …, *x_i7_*. The random coefficients of the *l*’th transect *t_kl_* in the *k*’the season and for the *m*’th date *d_m_* were normally distributed with a mean of zero.

**Eliminating** **seasonal interaction effect for sheep and goats:**

${ln(\mu}_{ijklm})= \eta_{ijklm}=\alpha{+ g}_{j}+s_{k}+\beta_{1}\times x_{i1}{+ \beta_{2}\times x_{i2}+\gamma}_{2k}\times x_{i2}+{\beta_{3}\times x_{i3}+\gamma}_{3k}\times x_{i3}+{\beta_{4}\times x_{i4}+\gamma}_{4k}\times x_{i4}+{\beta_{5}\times x_{i5}+\gamma}_{5k}\times x_{i5}+{\beta_{6}\times x_{i6}+\gamma}_{6k}\times x_{i6}+\beta_{7}\times x_{i7}+t_{kl}+d_{m}, ( 2)$

**Eliminating main effect for sheep and goats;**

${\ln(\mu}_{ijklm})= \eta_{ijklm}=\alpha{+ g}_{j}+s_{k}+\beta_{1}\times x_{i1}{+ \beta_{2}\times x_{i2}+\gamma}_{2k}\times x_{i2}+{\beta_{3}\times x_{i3}+\gamma}_{3k}\times x_{i3}+{\beta_{4}\times x_{i4}+\gamma}_{4k}\times x_{i4}+{\beta_{5}\times x_{i5}+\gamma}_{5k}\times x_{i5}+{\beta_{6}\times x_{i6}+\gamma}_{6k}\times x_{i6}+t_{kl}+d_{m,} (3)$

**Eliminating seasonal interaction effects for cattle**:

${\ln(\mu}_{ijklm})= \eta_{ijklm}=\alpha{+ g}_{j}+s_{k}+\beta_{1}\times x_{i1}{+ \beta_{2}\times x_{i2}+\gamma}_{2k}\times x_{i2}+{\beta_{3}\times x_{i3}+\gamma}_{3k}\times x_{i3}+{\beta_{4}\times x_{i4}+\gamma}_{4k}\times x_{i4}+{\beta_{5}\times x_{i5}+\gamma}_{5k}\times x_{i5}+\beta_{6}\times x_{i6}+t_{kl}+d_{m,}$ (4)

**Eliminating main effect for cattle:**

${\ln(\mu}_{ijklm})= \eta_{ijklm}=\alpha{+ g}_{j}+s_{k}+\beta_{1}\times x_{i1}{+ \beta_{2}\times x_{i2}+\gamma}_{2k}\times x_{i2}+{\beta_{3}\times x_{i3}+\gamma}_{3k}\times x_{i3}+{\beta_{4}\times x_{i4}+\gamma}_{4k}\times x_{i4}+{\beta_{5}\times x_{i5}+\gamma}_{5k}\times x_{i5}+t_{kl}+d_{m}, (5)$

**Eliminating seasonal interaction effects for elevation**:

${\ln(\mu}_{ijklm})= \eta_{ijklm}=\alpha{+ g}_{j}+s_{k}+\beta_{1}\times x_{i1}{+ \beta_{2}\times x_{i2}+\gamma}_{2k}\times x_{i2}+{\beta_{3}\times x_{i3}+\gamma}_{3k}\times x_{i3}+{\beta_{4}\times x_{i4}+\gamma}_{4k}\times x_{i4}+\beta_{5}\times x_{i5}+t_{kl}+d_{m}, (6)$

**Eliminating main effect for elevation:**

${\ln(\mu}_{ijklm})= \eta_{ijklm}=\alpha{+ g}_{j}+s_{k}+\beta_{1}\times x_{i1}{+ \beta_{2}\times x_{i2}+\gamma}_{2k}\times x_{i2}+{\beta_{3}\times x_{i3}+\gamma}_{3k}\times x_{i3}+{\beta_{4}\times x_{i4}+\gamma}_{4k}\times x_{i4}+t_{kl}+d_{m}, (7)$
